# Association of prior lymphopenia with mortality in pneumonia: a cohort study in UK primary care

**DOI:** 10.3399/bjgp20X713981

**Published:** 2021-01-26

**Authors:** Fergus Hamilton, David Arnold, Rupert Payne

**Affiliations:** Population Health Sciences, University of Bristol, Bristol; microbiology registrar, Department of Infection Science, North Bristol NHS trust.; Population Health Sciences, University of Bristol, Bristol; respiratory registrar, Academic Respiratory Unit, North Bristol NHS Trust, Bristol.; Centre for Academic Primary Care, Bristol.

**Keywords:** biomarker, infections, lymphopenia, pneumonia, primary care, respiratory

## Abstract

**Background:**

Lymphopenia (reduced lymphocyte count) during infections, such as pneumonia, is common and is associated with increased mortality. Little is known about the relationship between lymphocyte count before developing infections and mortality risk.

**Aim:**

To identify whether patients with lymphopenia who develop pneumonia have increased risk of death.

**Design and setting:**

A cohort study set in the Clinical Practice Research Datalink (CPRD) linked to national death records, in primary care. This database is representative of the UK population and is extracted from routine records.

**Method:**

Patients aged >50 years with a pneumonia diagnosis were included from January 1998 until January 2019. The relationship between lymphocyte count and mortality was measured, using a time-to-event (multivariable Cox regression) approach, adjusted for age, sex, social factors, and potential causes of lymphopenia. The primary analysis used the most recent test before pneumonia. The primary outcome was 28-day, all-cause mortality.

**Results:**

A total of 40 909 participants with pneumonia were included, with 28 556 having had a lymphocyte count test before pneumonia (median time between test and diagnosis was 677 days). When lymphocyte count was categorised (0–1 × 10^9^ cells/L, 1–2 × 10^9^ cells/L, 2–3 × 10^9^ cells/L, >3 × 10^9^ cells/L, never tested), both 28-day and 1-year mortality varied significantly: 14%, 9.2%, 6.5%, 6.1%, and 25%, respectively, for 28-day mortality, and 41%, 29%, 22%, 20%, and 52% for 1-year mortality. In multivariable Cox regression, lower lymphocyte count was consistently associated with increased hazard of death.

**Conclusion:**

Lymphopenia is an independent predictor of mortality in primary care pneumonia. Even low–normal lymphopenia (1–2 × 10^9^ cells/L) is associated with an increase in short- and long-term mortality compared with higher counts.

## INTRODUCTION

Lymphopenia (a reduction in the normal concentration of lymphocytes) has long been implicated as a potential biomarker for acute infection; many studies show that lymphopenia during, or at the start of, infection is associated with poor outcomes.^1^^–^^4^

Exploring the mechanisms and therapeutic implications of this phenomenon has been the subject of recent reviews, and remains largely unexplained.^5^^,^^6^

Recent work has also shown that lymphopenia may predict infection, and infection-related mortality, even when the lymphocyte count test significantly precedes the infection, with some studies supporting an increased risk of a wide range of infections (including pneumonia) and infection-related mortality, though no work has been performed in the broader primary care population with pneumonia.^7^^,^^8^

Respiratory infections such as pneumonia are very common in primary care,^9^ and greater understanding of predictive features would be of significant value to clinicians.

In this study the effect of lymphocyte counts on mortality was estimated in a large, unselected, primary care population with primary-care coded pneumonia.

## METHOD

This cohort study was performed in the Clinical Practice Research Datalink (CPRD), a large, representative UK primary care database, which holds coded primary care electronic health records, including blood tests, prescriptions, diagnostic codes, and immunisations. Of the English practices in this database, 70% are linked to the Office for National Statistics (ONS) mortality data; a national death registry.

Analysis was conducted and reported in line with the STROBE guidance (see Supplementary Box S1).

### Participants

Participants in this study were included from January 1998 until January 2019, had a coded diagnosis of pneumonia, and were aged >50 years at the time of pneumonia diagnosis. No other clinical exclusion criteria were used. CPRD records were included if they were considered ‘up to standard’ and of ‘acceptable’ quality (both internal CPRD metrics) for 6 months before a pneumonia diagnosis. If participants had multiple pneumonia episodes, the first episode was the one used.

### Outcomes

The primary outcome in this study was 28-day mortality, with a secondary outcome of 1-year mortality following pneumonia diagnosis.

### Lymphopenia definition

Blood test data are automatically extracted from GP medical records into the CPRD, with tests performed on a variety of platforms, across multiple laboratories in the UK. All laboratory results are used for routine clinical work, and there is likely to be limited relevant laboratory variation for this assay. The result and date of the test were extracted, and extreme values (lymphocyte count >10 × 10^9^ cells/L) were removed as outliers.

**Table:** How this fits in

Low lymphocyte levels during infection are common and associated with increased risk of death. This study, in a representative UK primary care population, shows that having a low lymphocyte count before acquiring an infection — even years before — is also associated with an increased risk of dying.

As most patients had multiple lymphocyte tests, several strategies were considered to define lymphocyte count for the subsequent analysis. The primary analysis was simply the most recent count, with no restrictions on time from blood test until pneumonia. The authors arbitrarily categorised this into integer cut points (0–1 × 10^9^ cells/L, 1–2 × 10^9^ cells/L, 2–3 × 10^9^ cells/L, and >3 × 10^9^ cells/L) for ease of clinical interpretation in primary analysis.

Multiple other approaches for quantifying lymphocyte count were performed, with details in the sensitivity analysis shown later.

### Confounding variables

As lymphocyte counts and mortality from pneumonia are known to be related to many common patient-level factors, relevant confounders were extracted from the CPRD. These included specific medical comorbidities, smoking status, alcohol usage, corticosteroid use, age (at time of pneumonia diagnosis), sex, and area-based socioeconomic deprivation (index of multiple deprivation [IMD]).

Medical comorbidities that are known to impact on lymphocyte count or mortality from pneumonia were included: cancer, autoimmune disease, organ transplantation, stem cell transplantation, HIV disease, previous stroke, diabetes (any type), peripheral vascular disease, and previous myocardial infarction.

All comorbidities, except cancer, were defined based on any relevant diagnostic code recorded before having pneumonia. Any cancer code (excluding non-melanotic skin cancer) in the preceding 2 years was taken as evidence of cancer.

Smoking status was categorised into ‘never smoker’, ‘current smoker’, or ‘ex-smoker’ based on clinical codes, and alcohol use defined by the presence of a variety of diagnostic codes for increased alcohol consumption. In patients without any smoking-related codes, smoking status was coded as ‘unknown’.

Corticosteroid use was defined as the prescription of >2 issues of an oral corticosteroid over the last 12 months, irrespective of dose or duration of issue. Finally, calendar year was included as a continuous variable, as management of pneumonia and testing may have changed over the period of the study.

### Statistical approach

Initially, patients who had ever had lymphocyte testing were compared with those who had never been tested, to identify any biases related to testing within the dataset.

Subsequently, primary analysis was by Cox regression to calculate hazard ratios (HRs), adjusted for the aforementioned covariates. Both non-adjusted and adjusted Kaplan–Meier curves were generated, with the adjusted plots generated with the marginal approach allowing for differential weighting of subgroups.^10^

Lymphocyte count was treated as a categorical variable (cut at integer break points for ease of clinical interpretation). Only patients with a recorded result were included in the primary analysis. Age was modelled as a linear variable. Smoking status and IMD were modelled as categorical variables. All other covariates were treated as binary. Very rare variables (<50 occurrences) were removed. As a secondary analysis, and for the purposes of visualisation, a restricted cubic spline Cox model was generated using lymphocyte count as a continuous variable.

As ‘up-to-standard’ CPRD data are relatively complete, and diagnosis was defined by the presence of a code, missing data were relatively rare. As such, a complete case analysis was performed. Follow-up was censored at 1 year. All analysis was performed in R (version 3.6.1), using the packages ‘tidyverse’, ‘survival’, ‘broom’, ‘Hmisc’, and ‘survminer’, with table generation using ‘tidyverse’ and ‘gtsummary’.^10^^,^^11^

### Sensitivity analyses

As many patients had multiple tests, multiple alternative approaches were explored to define lymphocyte count, categorising lymphocyte count based on quartiles rather than integer cut points, and additionally using alternative choice of measurements, that is, excluding measurements >1 year before pneumonia diagnosis; excluding measurements <6 months before pneumonia diagnosis; using the first ever measurement; and selecting the maximum, minimum, and mean counts irrespective of time.

In order to consider the potential bias generated by testing, a subsequent Cox model including patients who had never had a lymphocyte count before pneumonia was generated.

Finally, length of time between the most recent test and pneumonia (time-to-event approach) was included as an interaction term with lymphocyte count.

### Data sharing

The Medicines and Healthcare products Regulatory Agency do not allow sharing of raw CPRD data. If access is required, Independent Scientific Advisory Committee can be contacted to generate similar datasets.

## RESULTS

### Participant demographics

A total of 40 909 participants were included in this study. Participant demographics are shown in Table 1, comparing patients who had ever had a lymphocyte count test (*n* = 35 690) with participants never tested (*n* = 5219).

**Table 1.** Participant demographics, *N* = 40 909

**Characteristic**	**Never tested, *n*, %^a^ (*n* = 5219)**	**Tested, *n*, %^a^ (*n* = 35 690)**	***P*-value^b^**
Age, years, median (IQR)	81 (69–88)	76 (66–85)	<0.001

Sex, female	2740 (52.5)	18 020 (50.5)	0.007

**Comorbidities**			
Diabetes	421 (8.1)	5404 (15.1)	<0.001
HIV status	2 (<0.1)	22 (0.1)	0.8
Ischaemic heart disease	829 (15.9)	7093 (19.9)	<0.001
Ischaemic stroke	1030 (19.7)	5337 (15.0)	<0.001
Alcohol excess	98 (1.9)	834 (2.3)	0.043
Autoimmunity	505 (9.7)	6054 (17.0)	<0.001
Corticosteroid user	467 (8.9)	5134 (14.4)	<0.001
Peripheral vascular disease	227 (4.3)	1844 (5.2)	0.013
Stem cell transplant	3 (0.1)	17 (<0.1)	0.7
Solid organ transplant	9 (0.2)	130 (0.4)	0.036
Cancer (solid organ)	224 (4.3)	1692 (4.7)	0.2
Haematological cancer	36 (0.7)	393 (1.1)	0.008

**Smoking status**			<0.001
Never smoker	1594 (30.5)	8021 (22.5)	—
Ex-smoker	920 (17.6)	16 165 (45.3)	—
Current smoker	467 (8.9)	3368 (9.4)	—
Unknown	2238 (42.9)	8136 (22.8)	—

**Mortality**			
28 day	1318 (25.3)	2601 (7.3)	<0.001
1 year	2721 (52.1)	8377 (23.5)	<0.001

a Unless stated otherwise.

b Statistical tests performed: Wilcoxon rank-sum test; χ^2^ test of independence; Fisher’s exact test. IQR = interquartile range.

Participants who had no blood tests recorded were generally slightly older than other participants, but with lower rates of all comorbidities. Remarkably, patients who had never had a lymphocyte test had a significant 1-year mortality of 52.1%, suggesting the tested group is significantly different to the non-tested one.

### Lymphocyte test timings and distributions

Across the 35 690 patients tested, 368 870 tests were performed. Patients had a median of 7 (interquartile range [IQR] 3–13) tests and the distribution of tests was highly skewed to the left. The maximum number of tests on one person was 287, though the vast majority of patients had far fewer.

**Figure 1.**
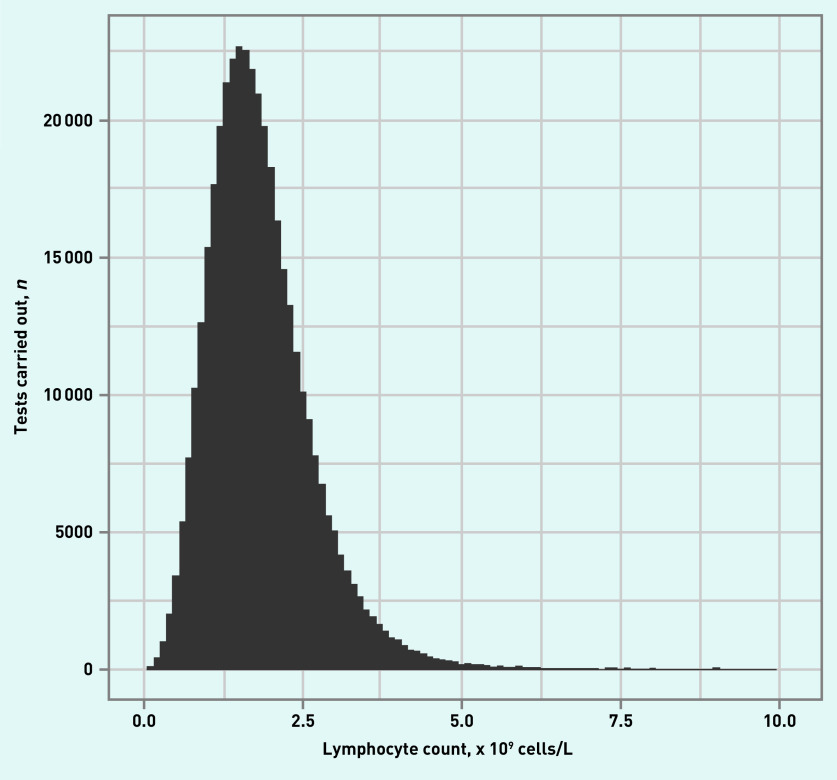
***Distribution of lymphocyte counts across all tests (N = 368 870 tests) across whole population.***

Testing steadily increased before diagnosis, with the peak number of tests just before pneumonia diagnosis (see Supplementary Figure S1). This was also highly skewed, with a median time from test of 677 days (IQR 10–1720). Lymphocyte test results were essentially normally distributed, with a mean (standard deviation [SD]) of 1.85 (0.88) × 10^9^ cells/L, as demonstrated in Figure 1.

There was no clear relationship between numbers of tests and lymphocyte count (see Supplementary Figure S2).

### Relationship between lymphocyte count and mortality

For the main analysis, the most recent lymphocyte count before pneumonia was chosen. This limited the sample size to 28 556 patients, as 7124 participants only had lymphocyte counts measured after, or on, the date of pneumonia diagnosis and were excluded. Of those 28 556 patients, 2544 (8.9%) met the primary outcome (died within 28 days), and 7962 (27.9%) met the secondary outcome (died within 1 year) (Table 2).

**Table 2.** Crude mortality for integer lymphocyte count cut off

**Outcomes**	**Lymphocyte count × 10^9^ cells/L, *n* (%)**			
	**0–1 (*n* = 3931)**	**1–2 (*n*= 14 963)**	**2–3 (*n*= 7419)**	**>3 (*n*= 2243)**
Primary: 28-day mortality	548 (13.9)	1375 (9.2)	485 (6.5)	136 (6.1)
Secondary: 1-year mortality	1606 (40.9)	4273 (28.6)	1627 (21.9)	456 (20.3)

**Figure 2.**
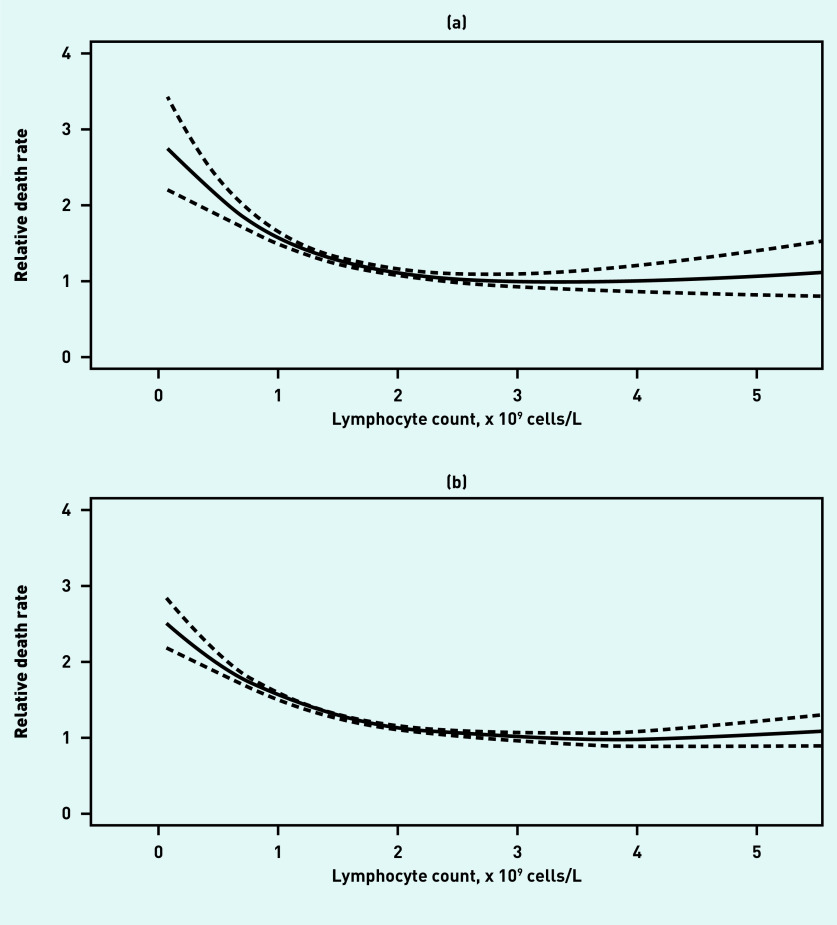
***Restricted cubic spline model showing relationship between lymphocyte count and mortality at (a) 28 days and (b) 1 year.***

There was a clear relationship between lymphocyte count and both outcomes. Figure 2 shows the relationship from the restricted cubic spline model, showing a non-linear increase in risk of mortality with decreasing lymphocyte count.

Crude mortality figures for each integer cut off are presented in Table 2, which show a clear decrease in risk with increasing lymphocyte count.

### Cox regression

Multivariable Cox regression was undertaken, with mortality censored at either 28 days (primary outcome) or 1 year (secondary outcome) (see Table 3). Due to low numbers of cases, HIV status and stem cell transplantation were removed from the Cox model.

**Table 3.** Adjusted Cox regression outputs for 28-day and 1-year mortality

**Characteristic^a^**	**28-day mortality**			**1-year mortality**		

	**HR**	**95% CI**	***P*-value**	**HR**	**95% CI**	***P*-value**
Age, years	1.06	1.06 to 1.07	<0.001	1.06	1.05 to 1.06	<0.001

Sex, female	1.00	0.93 to 1.09	>0.90	0.87	0.83 to 0.91	<0.001

**Lymphocyte count, × 10^9^ cells/L**						
0–1	1.63	1.44 to 1.84	<0.001	1.59	1.48 to 1.71	<0.001
2–3	ref	ref	—	ref	ref	—
1–2	1.15	1.03 to 1.27	0.009	1.14	1.08 to 1.21	<0.001
>3	0.95	0.78 to 1.15	0.6	0.91	0.82 to 1.01	0.069

**Comorbidities**						
Diabetes	0.99	0.89 to 1.11	0.90	1.07	1.01 to 1.14	0.027
Ischaemic heart disease	0.82	0.74 to 0.90	<0.001	0.98	0.93 to 1.03	0.40
Ischaemic stroke	1.40	1.28 to 1.54	<0.001	1.29	1.22 to 1.36	<0.001
Peripheral vascular disease	0.95	0.81 to 1.13	0.60	1.08	0.99 to 1.18	0.081
Alcohol excess	1.52	1.15 to 2.02	0.004	1.32	1.13 to 1.55	<0.001
Autoimmunity	0.87	0.77 to 0.97	0.010	0.92	0.87 to 0.98	0.005
Solid organ transplant	0.97	0.40 to 2.33	>0.90	1.14	0.76 to 1.70	0.50
Cancer (solid organ)	2.86	2.52 to 3.23	<0.001	2.98	2.77 to 3.20	<0.001
Haematological cancer	0.95	0.64 to 1.41	0.80	1.73	1.46 to 2.06	<0.001
Corticosteroid user	1.12	1.00 to 1.26	0.053	1.36	1.28 to 1.44	<0.001

**Smoking status**						
Never smoker	ref	ref	—	ref	ref	—
Ex-smoker	0.97	0.87 to 1.08	0.50	1.05	0.99 to 1.12	0.12
Current smoker	1.42	1.20 to 1.67	<0.001	1.48	1.35 to 1.62	<0.001
Unknown	1.43	1.28 to 1.60	<0.001	1.25	1.17 to 1.34	<0.001

**Calendar year**	0.95	0.94 to 0.96	<0.001	0.97	0.96 to 0.97	<0.001

a HIV status and stem cell transplant removed as few instances; Index of Multiple Deprivation not shown for brevity. HR = hazard ratio.

**Figure 3.**
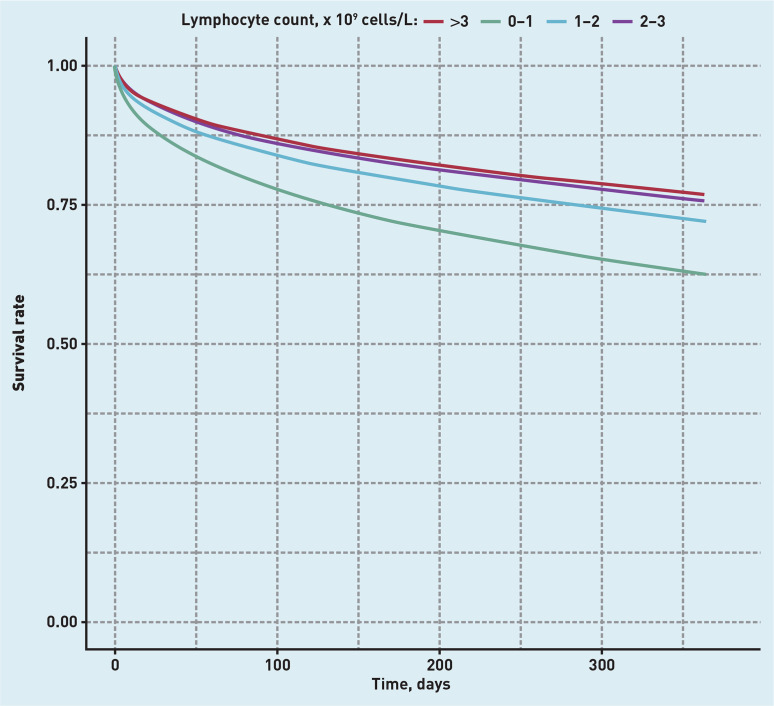
***Kaplan-Meier curves for survival from pneumonia at 1 year, stratified by lymphocyte count and adjusted for confounders.***

There was a clear association between having low lymphocyte count and increased risk of death at 28-days post-pneumonia, with an adjusted HR of 1.63 (95% confidence interval [CI] = 1.44 to 1.84) for lymphocyte counts <1 × 10^9^ cells/L compared to 2–3 × 10^9^ cells/L. A similar effect was observed for 1-year mortality (adjusted HR 1.59, 95% CI = 1.48 to 1.71). Unsurprisingly, most comorbidities were also associated with increased mortality, except autoimmunity and ischaemic heart disease, which were both associated with reduced mortality at 28 days and 1 year. Corresponding Kaplan-Meier survival curves are shown in Figure 3 (adjusted) and Supplementary Figure S3 (unadjusted).

### Sensitivity analyses

For the quartile model, full Cox regression outputs for the primary outcome are shown in Supplementary Table S1. Briefly, there remained a strong relationship with mortality, with the HR for death for those in the lowest quartile (0.09–1.31 x 10^9^ cells/L) of 1.56, 95% CI = 1.39 to 1.74. Mortality at 28 days was 13.9% and 6.1% in the lowest and highest quartiles of lymphocyte count, respectively (see Supplementary Table S2).

Abbreviated results (HR for lymphocyte count only) of models using alternative exposure definitions are presented in Supplementary Table S3. These associations were also maintained when the timing of testing was varied; the strongest association between lymphopenia and mortality was observed when only measurements in the past year were included (HR 1.57, 95% CI = 1.45 to 1.69 for lowest versus highest quartile of lymphocyte count), and the weakest association was observed when either the first measurement or mean measurement was used (HR 1.29, 95% CI = 1.21 to 1.37 for first measurement). The association was maintained (HR 1.37, 95% CI = 1.28 to 1.47) when measurements in the 6 months before pneumonia were excluded to ensure results were not directly related to the pneumonia itself.

Finally, the association persisted when the minimum, maximum, or mean lymphocyte count was used; the minimum count showed a stronger association (HR 1.59, 95% CI = 1.49 to 1.70). Unsurprisingly, the time between lymphocyte test and pneumonia varied significantly with each definition. In the ‘first ever’ approach, the median time from test until pneumonia was 1720 days (data not shown).

In summary, the association between alternative definitions of lymphocyte count and outcomes was maintained; however, the association was strongest when using lymphocyte tests that were closely temporally related to the pneumonia diagnosis, and when taking the minimum ever recorded lymphocyte count.

In the sensitivity analysis including patients who had never had a lymphocyte test (*n* = 5219) risk estimates were similar, with an HR of 1.66, 95% CI = 1.47 to 1.88 for a lymphocyte count of 0–1 x10^9^ cells/L compared to the reference (2–3 x 10^9^ cells/L) (see Supplementary Table S4).

There was no evidence from the interaction model that the strength of association between lymphocyte count and mortality was modified by time to test or number of tests.

## DISCUSSION

### Summary

In this large primary care cohort of patients with pneumonia, there was a clear relationship between low lymphocyte count and increased mortality. In particular, even mild lymphopenia, within the defined normal range (lymphocyte count 1–2 × 10^9^ cells/L), was associated with a significant increase in both short- and longer-term mortality.

Lymphopenia is robustly associated with short- and long-term survival in pneumonia, with increasing risk with lower lymphocyte counts. Patients with low–normal lymphocyte counts (1–2 x 10^9^ cells/L) are at significantly increased risk of mortality.

### Strengths and limitations

This study has many strengths. First, the large-scale cohort used a nationally representative UK primary care dataset, linked to mortality data. Second, the large number of study patients (*N* = 28 556) allowed for firm conclusions about risk estimates, and the quality of CPRD coding enabled testing for multiple covariates, none of which substantially altered the hazard estimate. Third, the wide variety of approaches to defining lymphocyte count provided reassurance that this is not simply a testing phenomenon.^12^ In particular, even definitions using relatively historic results (for example, first ever lymphocyte count) showed an association with subsequent pneumonia mortality.

This study has weaknesses consistent with similar observational analyses of large routine datasets. In particular, the authors did not have information on why tests were performed, though an interaction between number of tests and the outcome was not found. It is also important to note that around 15% of patients did not have a recorded test, and this population was quite different to the tested population, with a 52.1% 1-year mortality. Second, though a broad range of confounding variables were included, it is possible that other causes for lymphopenia were not recorded, for example, moderate alcohol intake not reported to GP. Despite this, unadjusted and adjusted risk estimates were broadly similar. Third, the present cohort comprised patients aged >50 years at the time of pneumonia diagnosis, and it is therefore necessary to be cautious about extrapolating findings to younger individuals.

### Comparisons with existing literature

There has been significant research interest in lymphopenia as a marker of poor immune function, though much work is pre-clinical and focuses on potential mechanisms of immune dysfunction.^5^ It remains unclear whether lymphopenia represents chronic failure of the immune system, or simply dysregulation. Some studies have found significant apoptosis of lymphocytes after infection, and this perhaps reflects dysregulation of immune control, rather than failure to maintain lymphocyte counts.^5^^,^^13^ It is also well established that lymphopenia occurs in critical illness, and it may be that lymphopenia simply represents an epiphenomena of sickness, though adjustment for other markers of sickness in the present study was made.^7^

In clinical data, one Oxford-based cohort study of adult emergency admissions showed lymphopenia to be an independent predictor of bacteraemia, though with modest performance (area under the curve 0.63).^1^ In a large, Danish population study of 98 344 invited individuals in Copenhagen, lymphopenia (defined as <1.1 × 10^9^ cells/L) was associated with a significant increase in infection and infection-related death (adjusted HR 1.41, 95% CI = 1.28 to 1.56 for any infection; HR 1.70, 95% CI = 1.37 to 2.10 for infection-related death).^8^ Of note, only 2352 (approximately 3%) of individuals had lymphopenia at this examination by their definition, and this was an invited population study, hence mortality was much reduced compared with the present study (5636 deaths [5.7%] at median follow up of 6 years). Of note, in contrast to the present study, increased mortality was not associated with low–normal lymphopenia (1–2 × 10^9^ cells/L). Another recent US study on a large invited cohort of 31 178 (the National Health and Nutrition Examination Survey) individuals found a relationship between lymphopenia and mortality, with a multivariable adjusted HR of 1.8, 95% CI = 1.6 to 2.1 for all-cause mortality with severe lymphopenia (lymphocyte count <1 × 10^9^ cells/L).^14^ Of note, this also relied on a single measure, and only half of patients included had linked mortality data, raising concern for bias.

Other work has focused on lymphopenia after diagnosis in infection as a poor prognostic marker,^2^^,^^3^^,^^15^ with one study on pneumonia (participants, *N* = 3043) finding lymphopenia as a predictor of early but not late mortality, in contrast to the present findings.^3^ In another study,^15^ severe lymphopenia (<0.724 x 10^9^ cells/L) was tested as an addition to CURB-65, a prognostic tool for pneumonia,^16^ modestly improving the predictive performance (C-statistic increased from 0.722 to 0.739) in a validation cohort.

In both studies set in secondary care, lymphopenia may represent acute illness, in contrast to the present study’s setting in primary care, where lymphopenia is more likely to be chronic.^4^

As far as the authors are aware, no studies have been performed in primary care looking at lymphopenia with a focus on pneumonia, which has significant implications. The vast majority of patients with respiratory tract infection present to primary care, and risk stratification here is critical. Further, the potential for identification and potential therapeutics is greater than in secondary care, as discussed in the next section.

### Implications for research and practice

This present study suggests lymphopenia is associated with both short- and long-term mortality. This has implications for risk stratification and for future therapeutics. For risk stratification, the authors suggest that even low–normal lymphopenia should be considered as a part of triage in identifying those likely to die. Further studies should evaluate this with other markers and scores for predicting outcomes in pneumonia, such as CURB-65. Alongside this, the role of lymphopenia in predicting occurrence and outcome of infection should be studied. Finally, the dramatic 1-year mortality (52.1%) in the non-tested group provides further evidence that indications for testing can strongly alter the predictive value of testing; that is a clinician decision to perform a test in itself can be predictive of mortality, thereby altering the predictive ability of that test; and clinicians should take this into account when interpreting this study.

For therapeutics, researchers should focus on identifying whether this is an epiphenomenon, or represents an opportunity to intervene in a dysregulated immune system. In particular, more accurate characterisation of lymphocyte functionality in pneumonia would be of significant value in assessing whether this is a therapeutic target.
